# Effect of climate conditions on the free and glycoside-derived volatile compounds in tomato cultivars

**DOI:** 10.5511/plantbiotechnology.25.0717a

**Published:** 2025-12-25

**Authors:** Yingtao Li, Yusuke Kamiyoshihara, Yonathan Asikin, Denise Tieman, Harry Klee, Miyako Kusano

**Affiliations:** 1 Degree Programs in Life and Earth Sciences, University of Tsukuba, Tsukuba, Ibaraki 305-8572, Japan; 2 College of Bioresource Sciences, Nihon University, Fujisawa, Kanagawa 252-0880, Japan; 3 Department of Bioscience and Biotechnology, Faculty of Agriculture, University of the Ryukyus, Nishihara, Okinawa 903-0213, Japan; 4 United Graduate School of Agricultural Sciences, Kagoshima University, Kagoshima, Kagoshima 890-0065, Japan; 5 Department of Horticultural Sciences, University of Florida, Gainesville FL 32611, USA; 6 Faculty of Life and Environment Science, University of Tsukuba, Tsukuba, Ibaraki 305-8572, Japan; 7 Tsukuba-Plant Innovation Research Center, University of Tsukuba, Tsukuba, Ibaraki 305-8572, Japan; 8 RIKEN Center for Sustainable Resource Science, Yokohama, Kanagawa 230-0045, Japan

**Keywords:** climate conditions, free volatile, glycoside-derived volatile, SPME, tomato

## Abstract

Commercial tomatoes have been increasingly criticized for their declining aroma, driving a search for tomato cultivars with more robust aroma profiles. Volatile organic compounds (VOCs) in tomato fruits play a crucial role in determining their aroma potential. This study investigates tomato cultivars with desirable aroma profiles by examining the levels of free- and glycoside-derived VOCs across 13 cultivars over a three-year period using non-targeted VOC profiling. The analysis detected 41 free VOCs and 35 VOCs released from the glycoside-derived precursors (glycoside-derived VOCs). Principal component analysis of the annotated free and glycoside-derived VOCs revealed that year-to-year differences were more pronounced than cultivar-to-cultivar variations. Among the annotated VOCs, 18 compounds were classified as unique free VOCs, while 12 were unique glycoside-derived VOCs. In 2020, the cultivar Livingstone’s stone exhibited significantly higher levels of several key aroma compounds compared to other cultivars and the control, Ailsa Craig. These compounds are known for their low odor thresholds and positive contributions to the overall aroma. These findings suggested that the growth year may substantially influence VOC production in tomato fruits, particularly for specific cultivars or VOCs, likely due to variations in climatic conditions. Consequently, optimizing the release of glycoside-derived VOCs offers a promising strategy to enhance tomato fruit aroma. This approach would be most effective by focusing on key VOCs from specific cultivars, while also accounting for the significant influence of growing year.

## Introduction

Tomato (*Solanum lycopersicum*) is among the most widely consumed edible plants in the nightshade family (Solanaceae). Renowned for their high nutritional value and unique flavor, tomato fruits are a global dietary staple (Zhang et al. 2023). Quality of tomato fruits is evaluated based on various factors, including flavor compounds, nutritional content, processing suitability, and storage potential (Ilahy et al. 2019; Keabetswe et al. 2019). Tomato flavor is primarily determined by two components: taste and aroma (Mikkelsen 2005). Taste arises from sugars, organic acids, and umami compounds (such as glutamic acid) (Kaur et al. 2023). The balance of these components defines the sweetness, sourness, and savory notes of the fruit. In contrast, the aroma of tomatoes is derived from volatile organic compounds (VOCs). Over 400 VOCs have been identified in tomato fruits (Petro-Turza 1986), including aldehydes, alcohols, esters, and terpenes (Selli et al. 2014). These VOCs originate from metabolic pathways that include lipid breakdown, amino acid and carotenoid metabolism, and carbohydrate metabolism. In recent years, concerns have grown over the declining quality of commercial tomatoes, particularly the diminished aroma (Tieman et al. 2017). This decline is attributed to breeding efforts that prioritize traits important to producers, such as yield, size, and disease resistance, which have unintentionally compromised tomato aroma (Folta and Klee 2016). In contrast, heirloom tomatoes, having evolved without intensive hybridization, maintain a richer VOC profile and superior aroma.

In tomato, VOCs are formed and released during ripening through the enzymatic conversion of precursors within the intact fruit. Additionally, when tissue is disrupted, VOCs are released due to the interaction of enzymes and substrates within the cells. In fresh tomatoes, the aroma is primarily determined by approximately 30-key VOCs (Baldwin et al. 2004). A portion of the VOCs in tomato fruits exists as non-volatile, flavorless glycoconjugates, primarily in the form of *O*-β-D-glucosides or *O*-glycosides (Winterhalter and Skouroumounis 1997), commonly referred to as glycoside-derived VOCs. Unlike free VOCs, glycoside-derived VOCs are not usually directly detectable by the human sense of smell in fresh tomato fruits. These compounds are potential candidates as aroma reserves for tomato fruits (Baldwin et al. 2000). The common structural feature of glycoside-derived VOCs is a glucopyranosyl unit linked via a β-D-glucosidic linkage to an aglycone, which may include monoterpenes, C_11_/C_13_-norisoprenoids, benzene derivatives, or linear alcohols (Sarry and Gunata 2004). Acid and enzymatic hydrolysis are the primary methods for releasing VOCs from these glycosidic precursors (Aubert et al. 2003). VOCs released from glycosidically bound precursors (aglycones) are referred to “glycoside-derived VOCs”. Qualitative and quantitative analysis of glycoside-derived VOCs is essential for identifying tomato cultivars with superior aroma potential (Özkaya et al. 2018).

To date, limited research has addressed how the production of free and glycoside-derived VOCs in tomato fruits is influenced by growing year, particularly across multiple cultivars. To fill this gap, we analyzed the composition and content of free and bound VOCs in 13 tomato cultivars over a three-year period, using headspace solid-phase microextraction coupled with gas chromatography-mass spectrometry (HS-SPME-GC-MS), a method that offers advantages such as minimal sample preparation and high sensitivity (Pang et al. 2012; Yuan et al. 2022). This comprehensive VOC profiling provides valuable insight into year-dependent variability in tomato aroma traits and can support the selection of cultivars with enhanced aroma or greater aroma potential.

## Materials and methods

### Chemicals

Water used in the study was purified using a Millipore-Q system (Millipore Corp., Saint-Quentin, France). The following compounds were sourced from Sigma–Aldrich (Merck), (St. Louis, MO, USA): alkane standard solution (C8–C20), ethylenediaminetetraacetic acid (EDTA), EPA 524.2 fortification solution (fluorobenzene and 1,2-dichlorobenzene-*d*_4_). Methanol (LC-MS grade), NaCl (water analysis reagent), and CHCl_3_ (guaranteed reagent) from FUJIFILM Wako Pure Chemical Corporation (Osaka, Japan). Rapidase Revelation Aroma was purchased from Oenobrands (Montpellier, France).

### Plant materials

The tomato accessions utilized in this study (Supplementary Table S1) were cultivated in an open field at Nihon University (Fujisawa, Kanagawa, Japan) consistent cultivation conditions throughout the three-year study period. Fully ripe fruits were harvested in July in 2020, 2021, and 2022, respectively. After the removal the seeds and gel, the fruit of each cultivar was diced into approximately 1 cm in size. The samples were frozen and shipped to the University of Tsukuba at approximately temperatures of −15°C. The samples were stored at −80°C until use.

### Extraction of free VOCs

Free VOCs in the tomato samples were extracted using an SPME fiber with a 50/30 µm coating of divinylbenzene/Carboxen/Polysimethylsiloxane (Sigma–Aldrich (Merck)) and subsequently analyzed by GC–time-flight-of–MS (LECO PEGASUS III, St. Joseph, MI, US) (Kusano et al. 2016). Briefly, the frozen tomato powder (3 g) was crushed using a Multi-beads Shocker (YASUI KIKAI Co., Ltd., Osaka, Japan) at 2800 rpm for 16 s. The procedure was conducted at room temperature (approx. 22–25°C). To this, an internal standard solution EPA 524.2 (200 µg l^−1^ for each component, 10 µl) an aqueous NaCl solution (1 ml, 20% w/w), and an EDTA solution (1 ml, 0.1 M, pH 7.5) were added to a 20 ml glass vial (Agilent Technologies, Santa Clara, MA, USA). The sealed vial, sealed with a screw cap (Agilent Technologies, Santa Clara, MA, USA), was then heated and shaken for 10 min at 50°C using a PAL autosampler (CTC Analytics AG, Zwingen, Switzerland). Three biological replicates were used for samples in 2020 and 2021, and four replicates in 2022.

### Extraction and hydrolysis of glycoside-derived VOCs

Glycoside-derived VOCs in tomato samples were extracted using the solid-phase extraction (SPE) technique (Kamiyoshihara et al. 2016). Frozen tomato tissue was lyophilized using an a lyophilizer (FDU-2110, Tokyo Rikakikai Co., Ltd. (EYELA), Tokyo, Japan) at −80°C for 48 h, then crushed under liquid nitrogen using a Multi-beads Shocker at 2800 rpm for 16 s (YASUI KIKAI, Osaka, Japan). Five grams of dried tomato powder was mixed with 20 ml of Milli-Q water. Next, 2 ml of CHCl_3_ was added, and the mixture was centrifuged at 4°C, 1200×g for 5 min. The first supernatant was collected, and 3 ml of Milli-Q water was added to the remaining residue. After re-suspension, the mixture was centrifuged again under the same conditions. The second supernatant was collected, and the two supernatants were combined to yield the glycoside-derived VOC fraction.

The resulting supernatant was loaded onto an Oasis HLB 3cc Vac cartridge (60 mg sorbent, Waters Corporation, Milford, MA, USA), the target compounds were then fractionally eluted, first with 6 ml of 50% aqueous methanol and subsequently with 6 ml of methanol. The fraction containing glycoside-derived VOCs was evaporated to dryness using a SpeedVac Concentrator (SPD1010, Thermo Fisher Scientific, Waltham, MA, USA) at 25°C under vacuum.

The dried extract was dissolved in 500 µl of 0.1 M citric buffer (pH 4.5) and combined with 100 µl of Rapidase® Revelation Aroma solution (20 mg ml^−1^ in citric buffer, β-glucoside enzyme activity: 4200 U g^−1^). The mixture was enzymatically hydrolyzed at 30°C for 4 h. For the analysis of glycoside-derived VOCs, the resulting hydrolysate (600 µl) was placed in a 20 ml glass vial along with an aqueous NaCl solution (600 µl, 20% w/w) and the EPA 524.2 fortification solution (10 µl). The sample was then analyzed by HS-SPME-GC-MS using the same procedure described for the free VOCs. The sample preparation for glycoside-derived VOCs differed from that for free VOCs only in the omission of EDTA.

### GC-MS analysis

The injection temperature was set to 250°C, and the linear velocity of the helium carrier gas flowed at a linear velocity of 15 ml min^−1^ into an Rxi-5SilMS column (30 m×0.25 mm i.d., film thickness: 0.25 µm, RESTEK, Bellefonte, PA, US). The column temperature was initially set to 40°C for 4 min, then gradually increased to 200°C at a rate of 10°C min^−1^, where it was held for 4 min. Mass spectra for compounds were obtained using a GC 6890N system coupled with a PEGASUS III MS (LECO). All raw data of detected metabolites were transferred from the ChromaTOF software in NetCDF format to MATLAB software 6.5 (MathWorks, Natick, MA, USA). The chromatograms were preprocessed using the high-throughput data analysis (HDA) method (Jonsson et al. 2005) and normalized using an internal standard, 1,2-dichlorobenzene-*d*_4_. The resolved MS spectra were matched against reference of mass spectra using the NIST mass spectral search program (version 2.2) for the NIST/EPA/NIH mass spectral library, and our custom software for peak annotation written in JAVA. Peaks were putatively annotated by comparing their resolved mass spectra and retention indices (RIs) to entries in several reference libraries. An annotation required a similarity score of 800 or greater. The libraries used for this comparison included the Adams library (3rd and 4th editions), the Terpenoids library, VocBinBase (Skogerson et al. 2011), NIST11, the FFNSC 3 Wiley Library, and our own custom library. RIs were calculated using an alkane standard solution (C8–C20) run under the same chromatographic conditions to improve reliability and the reproducibility of compound identification. Peak identification and annotation were based on retention indices and fragment ions, like the strategy proposed by Yuan et al. (2024), which integrated mass spectra and RI comparison for putative compound annotation.

### Statistical analysis

All data were recorded using Microsoft Office 2021. The average value and standard deviation (SD) for free- and glycoside-derived VOCs were calculated using Microsoft Excel 2021. Significant differences between each cultivar and the control cultivar, Alisa Craig, were evaluated by Student’s *t*-test. Multivariate analysis of the free- and glycoside-derived VOCs emitted by the 13 tomato cultivars was performed using SIMCA 14 software (Umetrics, Swedish). *Z*-scores were used to standardize the data for free- and glycoside-derived VOCs using Microsoft Office 2021. Heatmap plots were generated using TBtools software (Chen et al. 2023).

## Results

### Composition of free- and glycoside-derived VOCs in the 13 tomato cultivars

**Figure 1:**
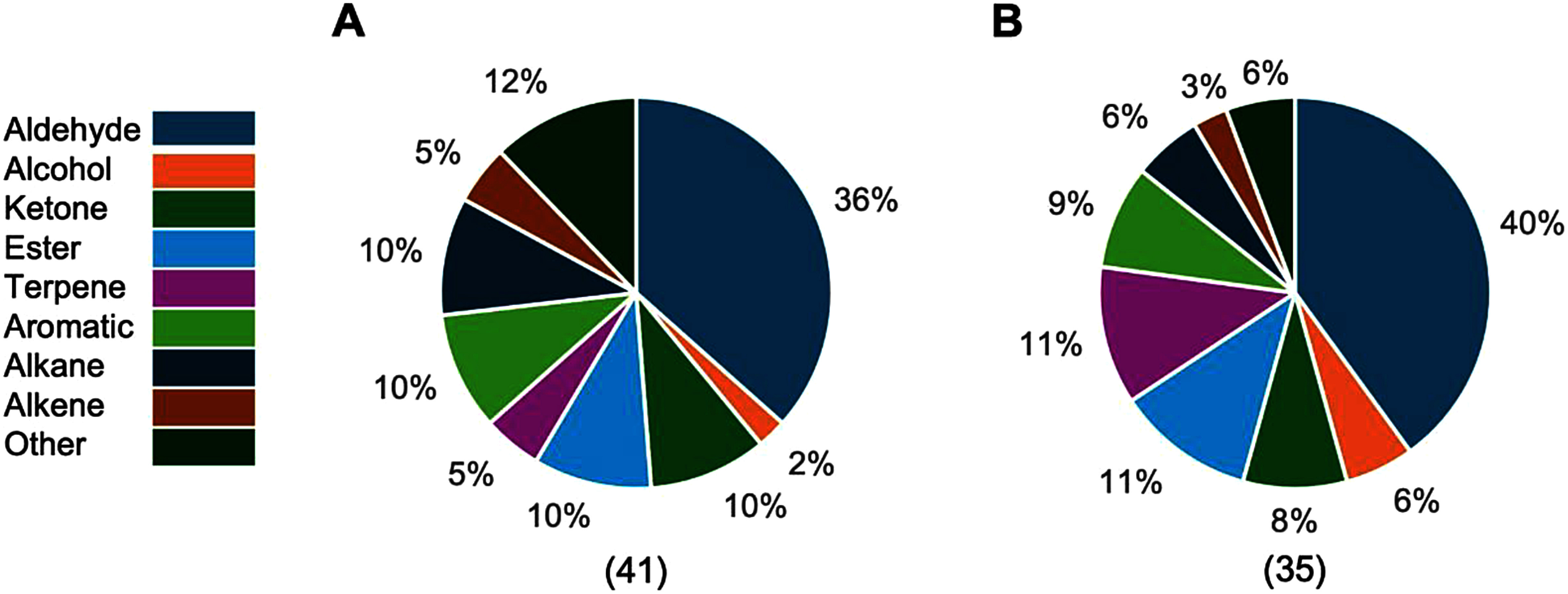
Figure 1. Percentage of annotated free (A) and glycoside-derived (B) VOCs in 13 tomato cultivars. The number of annotated free- and glycoside-derived VOCs is shown in parentheses. The percentage represents the proportion of this class of VOC among the annotated free- and glycoside-derived VOCs. 100% corresponds to 41 (free) and 35 (glycoside-derived) annotated VOCs.

The 13 tomato cultivars analyzed in this study exhibit diverse flavors and colors, encompassing a broad range of flavor profiles. To evaluate whether identical field conditions influenced VOC accumulation, the 13 tomato cultivars were cultivated from 2020 to 2022. Free- and glycoside-derived VOCs were analyzed using SPME-GC-MS analysis (Supplementary Figure S1). Forty-one of free VOC compounds were categorized as 15 aldehydes, four esters, four ketones, two terpenes, four aromatic compounds, one alcohol, four alkanes, two alkenes, and five other compounds in the 178 detected peaks. In contrast, 35 of glycoside-derived VOCs were annotated, consisting of 14 aldehydes, four esters, three ketones, four terpenoids, three aromatic compounds, two alcohols, two alkanes, one alkene, and two other compounds in 134 detected peaks. The proportion of each compound class within the annotated free- and glycoside-derived VOCs is illustrated in Figure 1. Aldehydes were the most prevalent in both free and glycoside-derived VOCs, comprising over 30% of the annotated compounds in each group. Furthermore, the proportions of ketones, esters, and alkanes were similar across free- and glycoside-derived VOCs. Several aldehydes, ketones, and other non-hydroxylated compounds were detected among the glycoside-derived VOCs, despite their limited propensity for glycosidic conjugation (Supplementary Table S2). This may be attributed to the presence of non-glycosidic VOC precursors in the SPE extracts and the use of AR2000, a complex enzyme mixture commonly applied in winemaking. In addition to β-glucosidase, AR2000 likely contains other enzymatic activities capable of hydrolyzing alternative precursors, thereby releasing non-glycosidic VOCs (Martínez et al. 2025; Wang et al. 2023).

### Distribution and changes of free- and glycoside-derived VOCs over three years

**Figure 2:**
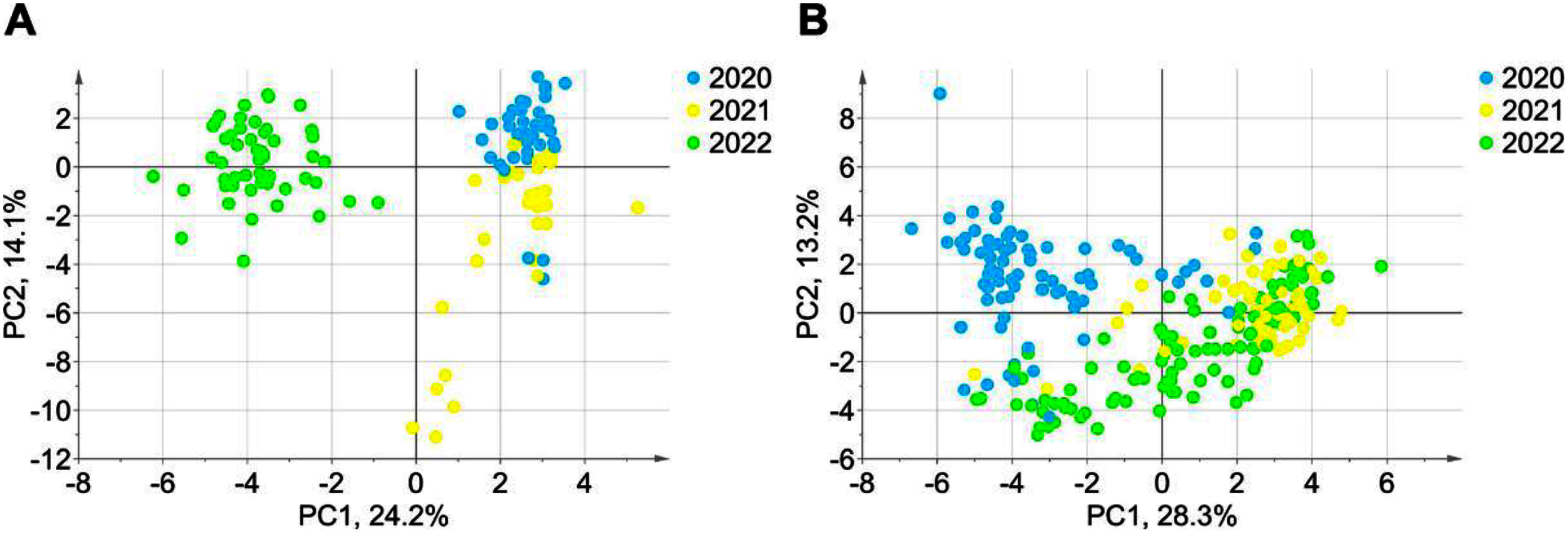
Figure 2. PCA score plot (A) of annotated compounds for free VOCs. PCA score plot (B) of annotated compounds for glycoside-derived VOCs. Data were obtained from GC-MS analysis of tomato samples grown in different years (*n*=3–4).

The distribution and temporal changes of annotated free- and glycoside-derived VOCs in the 13 tomato cultivars over three years were visualized using principal component analysis (PCA) score scatter plots and *Z*-score heatmaps (Figure 2, Supplementary Figure S2). In the PCA score scatter plots (Figure 2A, B), the free VOC profiles from 2022 exhibited a distinct separation from those of other years along the first principal component (PC1). For the glycoside-derived VOCs, the 2020 samples were distinctly separated along PC2, while samples from the other years showed no clear differentiation along this axis. In contrast to these strong year-dependent effects, no significant differences were observed among the 13 cultivars in either the free or glycoside-derived VOC profiles across the three-year period.

To explore the year-to-year changes in individual VOCs, *Z*-score clustering analysis was performed (Supplementary Figure S2, Supplementary Data S1). The heatmaps revealed that each cultivar possessed a distinct VOC profile, with considerable variation observed across the three-year study period. For instance, while the free VOC profile of Livingstone’s stone remained relatively stable over the three years, it exhibited significantly higher levels of 17 glycoside-derived VOCs specifically in 2020. In contrast, other cultivars showed major variations in their free VOCs. For instance, Sungold showed elevated levels of 11 free VOCs in 2020, while Chika showed elevated levels of 26 free VOCs in 2021, compared to the average of all cultivars.

### Comparison of common and unique VOCs between free- and glycoside-derived VOCs

**Figure 3:**
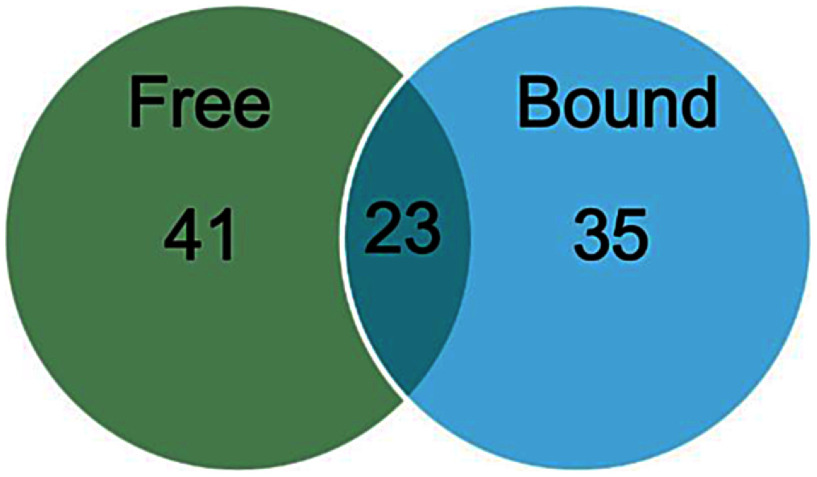
Figure 3. Venn diagram showing the number of annotated free- and glycoside-derived VOCs in the 13 tomato cultivars over three years.

Figure 3 shows the number of common- and specific compounds to free- and glycoside-derived VOCs in 13 tomato cultivars. A total of 23 annotated compounds were annotated in both free- and glycoside-derived VOCs (Supplementary Table S2). Among these common compounds, hexanal, 1-nitro-pentane, and methyl salicylate were known to be particularly abundant and to exhibit low odor thresholds (Özkaya et al. 2018). This suggests that these VOCs may play significant roles in shaping the aroma profile and potential of tomato fruits. In the free VOC profiles, 18 compounds were annotated as unique components (Supplementary Table S3). Among them, 6-methyl-5-hepten-2-one, and (*Z*)-2-heptenal, were recognized as major contributors due to their low odor thresholds (Baldwin et al. 2015). These compounds are likely critical contributors to the aroma profiles in free VOCs (Distefano et al. 2022). In case of the glycoside-derived VOC profiles, 12 compounds were exclusively detected (Supplementary Table S4). Notably, citronellol, neral, and geranial, which are associated with popular floral and fruity aroma with low odor thresholds, were detected. These compounds are considered to enhance the aroma potential of tomato fruits (Davidovich-Rikanati et al. 2007). This analysis highlights the importance of unique free- and glycoside-derived VOCs in uncovering novel aroma candidates.

### Comparison of free- and glycoside-derived VOC levels among the 13 cultivars

**Figure 4:**
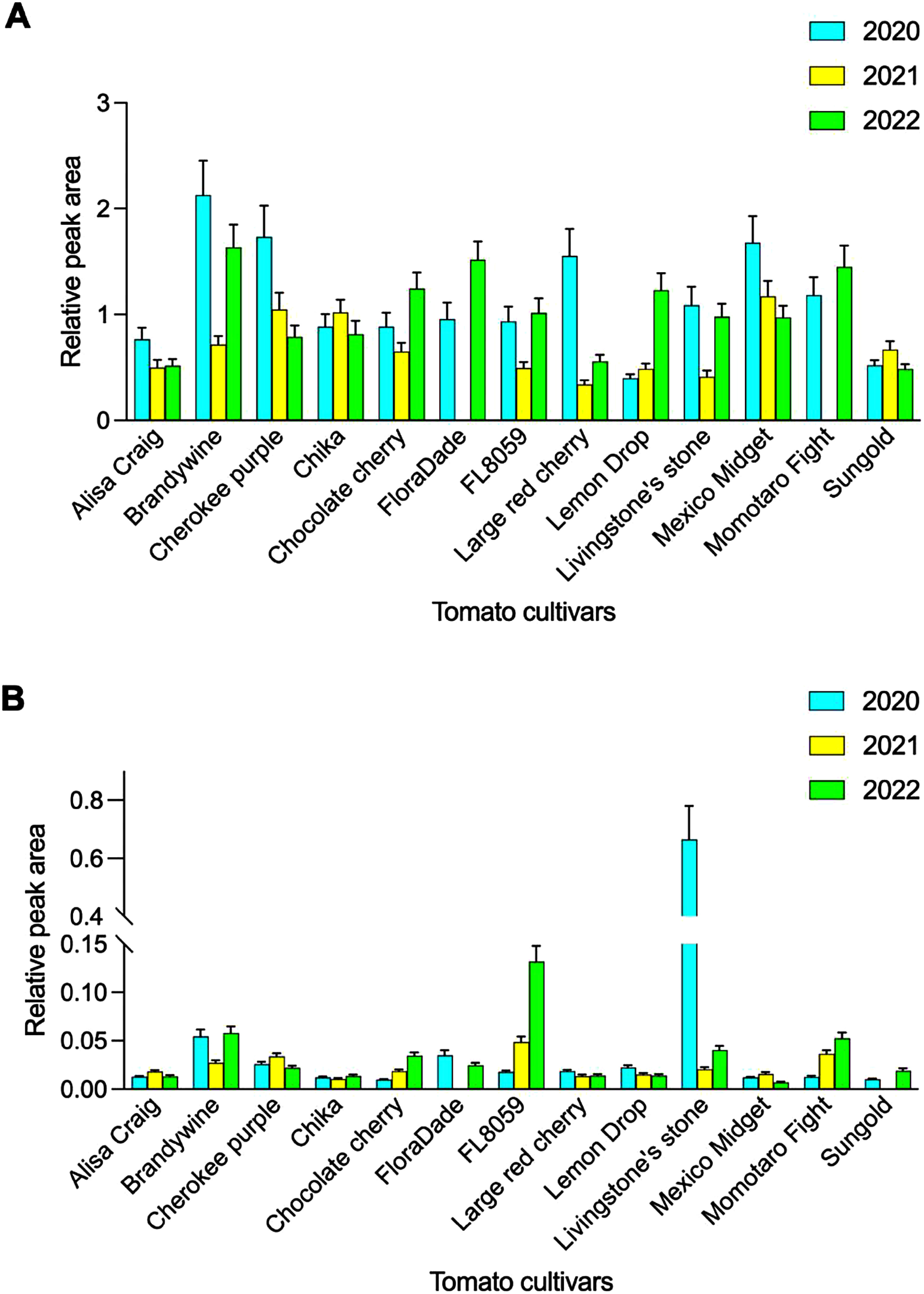
Figure 4. Comparison of the total normalized peak areas of unique free- (A) and glycoside-derived (B) VOCs in 13 tomato cultivars. In 2021, the missing data for FloraDade was due to cultivation pollution. Missing data for Momotaro Fight and Sungold were due to contamination in sample handling. The number of biological replicates, *n*=3 except for the 2022 samples, where *n*=4. Error bars indicate SD.

Figure 4 shows the total relative peak areas of free- and glycoside-derived VOCs detected in the 13 cultivars. Among these compounds, 18 and 12 were uniquely detected in free- and glycoside-derived VOCs, respectively. Across all tomato cultivars, the levels of free VOCs were higher than those in glycoside-derived VOCs being approximately 60 times higher than that of glycoside-derived VOCs. Accumulation of the free- and glycoside-derived VOCs varied significantly depending on both the cultivar and the growing year. In 2020, Livingstone’s stone showed a significantly higher level of specific glycoside-derived VOCs compared to the other cultivars. Among glycoside-derived VOCs, neral, citronellol, and geranial are known as critical contributors to the aroma potential of tomato fruits. Their low odor thresholds make them valuable for evaluating the aroma potential of tomato cultivars (Grosch 2001).

### Changes in the profiles of unique free- and glycoside-derived VOCs between Livingstone’s stone and Ailsa Craig in 2020

To explore potential aroma candidates, we analyzed the contributions of the unique glycoside-derived VOCs to the elevated accumulation observed in Livingstone’s stone harvested in 2020. The cultivar Ailsa Craig was selected as the control due to its excellent flavor (Hessayon 1997) and having been in cultivation for more than 100 years. This cultivar was widely used in breeding greenhouse tomato cultivars in the US and is in the pedigrees of many significant cultivars.

**Figure 5:**
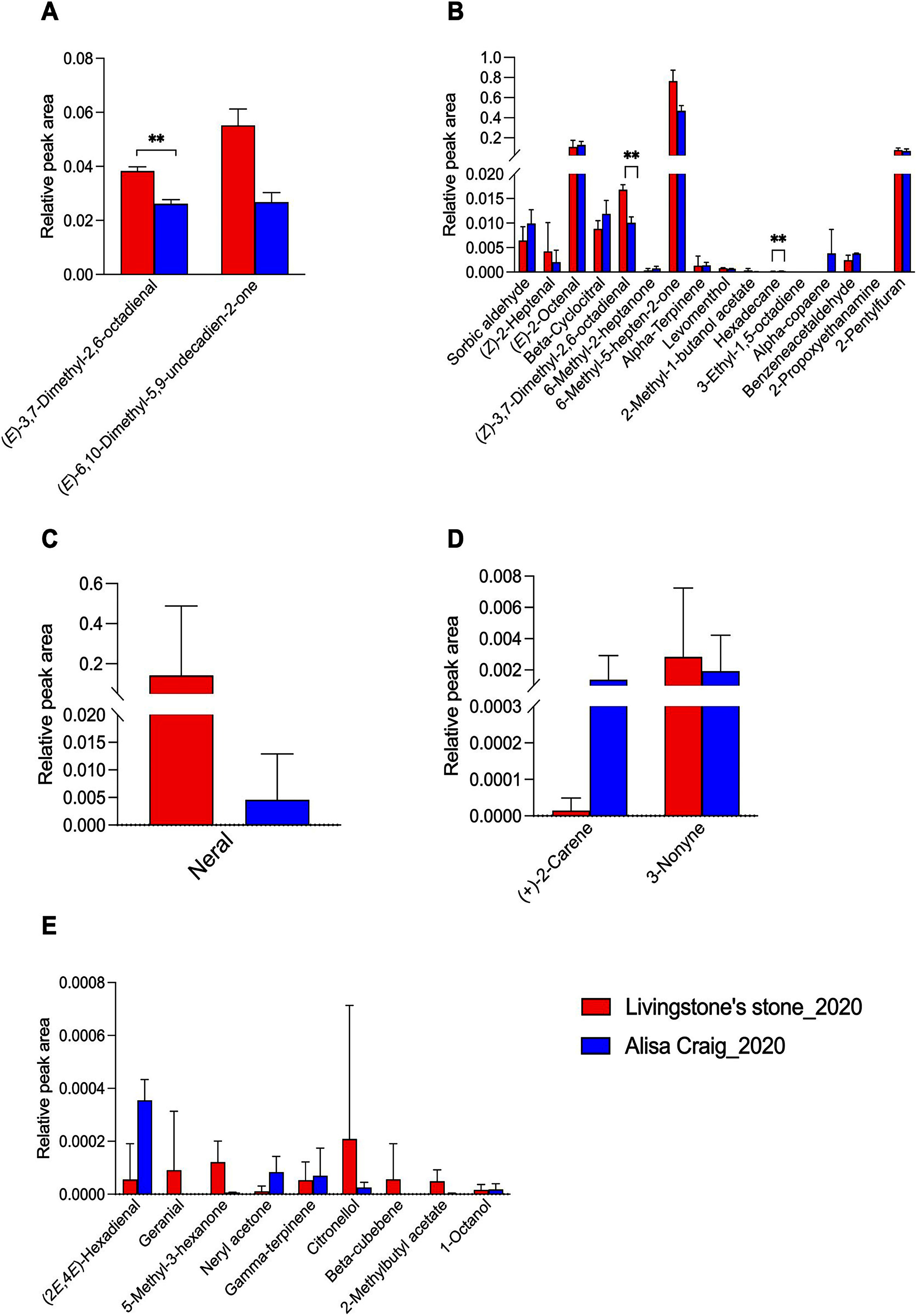
Figure 5. Comparison of the relative peak areas of unique free- (A, B) and glycoside-derived VOCs (C, D, E) in Livingstone’s stone and Alisa Craig cultivars grown in 2020. The number of biological replicates, *n*=3. Statistical analysis was performed using *t*-test (* *p*<0.05, ** *p*<0.005). Error bars indicate SD.

In 2020, total of 18 unique free VOCs were detected across both Livingstone’s stone and Ailsa Craig (Figure 5A, B). Among these, six compounds were present at higher levels in Livingstone’s stone, including (*Z*)-2-heptenal, (*Z*)-3,7-dimethyl-2,6-octadienal, 6-methyl-5-hepten-2-one, (*E*)-6,10-dimethyl-5,9-undecadien-2-one, levomenthol, and 2-propoxyethanamine. Significant differences in the levels of several key aroma compounds were observed between the two cultivars (Supplementary Table S6). In particular, (*Z*)-3,7-dimethyl-2,6-octadienal, (*Z*)-2-heptenal, and 6-methyl-5-hepten-2-one were among the most different. The latter two compounds are known for their low odor thresholds, making them important contributors to the perceived aroma profile. A similar trend was observed for unique glycoside-derived VOCs (Figure 5C, D, E). Of the 12 glycoside-derived VOCs identified in both cultivars, Livingstone’s stone exhibited higher levels of the seven glycoside-derived VOCs, including neral, geranial, 5-methyl-3-hexanone, citronellol, β-cubebene, 2-methylbutyl acetate, and 3-nonyne (Supplementary Table S7). Notably, neral and geranial have exceptionally low odor thresholds, and neral, in particular, exhibited the highest relative abundance among all annotated VOCs. Although 3-nonyne was also present at a relatively high level, its contribution to overall aroma remains uncertain due to the lack of a defined odor threshold. The distinctive profile of unique free- and glycoside-derived VOCs in Livingstone’s stone, particularly the enrichment of compounds with low odor thresholds, suggests a potential role in contributing to its unique aroma characteristics. These cultivar-dependent variations in VOC composition were especially evident in 2020.

## Discussion

### Impact of year- and cultivar-dependent differences in VOC levels in heirloom tomatoes

This study examined the effects of yearly variations on the free- and glycoside-derived VOC profiles of 13 tomato cultivars using HS-SPME-GC-MS. The results indicated that year-to-year differences had a greater impact on the VOCs profiles than cultivar-dependent differences. Some VOCs within the specific VOC group, such as neral and geranial, could be extracted as key contributors to the aroma potential of tomato cultivars due to their low odor thresholds and distinctive odors (Distefano et al. 2022). Notably, Livingstone’s stone showed the highest accumulation in glycoside-derived VOCs in 2020. However, the significant annual variation observed across all cultivars underscores the importance of conducting multiyear evaluations when screening for desirable aroma profiles. The distinct clustering patterns observed in the PCA (Figure 2, Supplementary Figure S3), further support our earlier observation that year-to-year variation exerts a strong influence on the production of VOCs in tomatoes. This variability was evident in both free- and glycoside-derived VOC profiles, suggesting that environmental factors likely affect multiple metabolic pathways involved in tomato VOC biosynthesis. This study also highlights the significant influence of yearly climatic variation on VOC production in tomatoes. The findings suggest that environmental factors play a more dominant role than previously understood, impacting both free- and glycoside-derived VOC content profiles across diverse cultivars. Some VOCs, such as (*Z*)-2-heptenal and 6-methyl-5-hepten-2-one, have low odor thresholds and characteristic aromas, and their levels exhibited year-to-year variation (Supplementary Figure S2). These compounds, previously identified by Baldwin et al. (2015) as key contributors to genotypic differences in tomato aroma, showed marked variability over three years in cultivars such as Cherokee purple, Brandywine and Chika. This result suggests that these VOCs may be particularly sensitive to environmental or temporal changes. The observed interannual variability in VOC content likely stems from climatic changes over the study period, as the tomatoes were grown in the same location but experienced significant variations in maximum temperature, minimum temperature, daylight duration, and precipitation (Supplementary Table S5). Among the climate factors examined, a distinct difference was observed in the daylight duration during July, when the tomato fruits were harvested: 70.8 h in 2020, compared to 191.6 h in 2021 and 195.6 h in 2022. There were six cultivars showing middle-size tomatoes used in this study (Ailsa Craig, Chika, Momotaro Fight, Livingston’s stone, and FloraDade) (Supplementary Table S1). Among them, the levels of the glycoside-derived VOCs, including neral, citronellol, and geranial, were especially increased in fruits of Livingstone’s stone harvested in 2020 (Figure 4, 5). It should be clarified why Livingstone’s stone showed sensitive to produce glycoside-derived VOCs by focusing on difference of daylight duration during harvesting period in near future. However, research on the effects of climate on other types of VOCs and glycoside-derived VOCs in tomato fruits remains limited. Further studies are needed to determine the relationship and underlying causes of climate-induced changes in VOCs in tomato fruits. This study highlights the importance of future research aimed at understanding the specific impacts of varying climate conditions on different classes of VOCs, including glycoside-derived VOCs, in tomato fruits. Such investigations will be crucial for developing strategies to enhance aroma and flavor profiles under changing environmental conditions.

### Integrating tomato cultivar and temporal influences on VOC profiles for predicting aroma potential

The numbers of specific compounds extracted in free- and glycoside-derived VOCs were comparable, at 18 and 12, respectively, primarily consisting of aldehydes, ketones, terpenes, and hydrocarbons (Supplementary Table S3, S4). However, the levels of specific free VOCs were significantly higher than specific glycoside-derived VOCs, consistent with findings reported by Ortiz-Serrano and Gil (2010). Unique VOC levels fluctuated unpredictably across cultivars and years, and no cultivar consistently exhibited either the highest or lowest accumulation throughout the three-year period. Unique VOC levels fluctuated unpredictably across cultivars and years, and no cultivar consistently exhibited either the highest or lowest accumulation throughout the three-year period. In contrast, the variability in the levels of unique glycoside-derived VOCs was less pronounced than that observed for the unique free VOCs. Notably, Livingstone’s stone exhibited substantially higher levels of unique glycoside-derived VOCs in its 2020 samples compared to other cultivars harvested in the same year. This suggests that Livingstone’s stone may possess greater aromatic potential under specific environmental conditions. The odor threshold, defined as the minimum concentration at which a human can detect a smell (Doty 2019), serves as a key indicator of a compound’s odor strength. A lower odor threshold signifies a more potent aroma, making VOCs with lower thresholds likely to be critical contributors to overall aroma perception. Among the unique VOCs identified in this study were several known aroma compounds. For example, (2*E*,4*E*)-hexadienal and 3-nonyne impart green aromas, while 2-methylbutyl acetate, neral, citronellol, and geranial contribute to fruity aromas. Notably, neral, and geranial were characterized by low odor threshold. Marković et al. (2007) showed that geraniol, citronellol, and neral positively influenced the aroma with floral and fruity notes, despite their low relative content in tomato fruits. Sitrit et al. (2008) showed that the content of geraniol and its derivatives (including geranial, neral, and citronellol) can be increased by the overexpression of specific genes, resulting in significantly altered the aroma profile of tomato fruits. Unlike free VOCs, glycoside-derived VOCs often require enzymatic activity to unleash their aromatic potential. This study using non-targeted VOC profiling of diverse tomato cultivars identified certain unique glycoside-derived VOCs with low odor thresholds that are enriched in specific cultivars in harvest year by applying appropriate conditions. It might imply that the aroma potential of tomato cultivars such as Livingstone’s stone is influenced by the environmental conditions during cultivation. These findings indicate that targeting glycoside-derived VOCs, particularly those found in tomatoes, could represent a novel strategy for enhancing tomato aroma through selective breeding or cultivation practices.

In conclusion, this study analyzed changes in free- and glycoside-derived VOCs from 2020 to 2022 for 13 tomato cultivars. Differences in the profiles of VOC between years were more pronounced than between cultivars in both free- and glycoside-derived VOCs. Particular tomato cultivars, such as Livingstone’s stone, exhibited significantly higher levels of specific glycoside-derived VOCs in 2020. This finding has important implications for the selection of environmentally sensitive cultivars with excellent aroma potential. Based on our study, the significance of glycoside-derived VOCs for tomato aroma can be further clarified in the future by investigating the extent of their contribution to tomato aroma potential through systematic quantitative analysis.

## Abbreviations

GC-MS: gas chromatography-mass spectrometry
HS-SPME: headspace solid-phase microextraction
SPE: solid-phase extraction
VOC: volatile organic compound

## Description of supplementary files

Supplementary Table S1. List of 13 tomato cultivars used in this study.

Supplementary Table S2. Commonly detected compounds annotated in both free- and glycoside-derived volatile organic compounds (VOCs) across 13 tomato cultivars over three years.

Supplementary Table S3. Specific compounds annotated in free volatile organic compounds (VOCs) across 13 tomato cultivars over three years.

Supplementary Table S4. Specific compounds annotated in glycoside-derived volatile organic compounds (VOCs) across 13 tomato cultivars over three years.

Supplementary Table S5. Main climate conditions from April to July in 2020–2022.

Supplementary Table S6. Relative peak areas of unique free volatile organic compounds (VOCs) in Livingstone’s stone and Ailsa Craig cultivars (2020–2022).

Supplementary Table S7. Relative peak areas of unique glycoside-derived volatile organic compounds (VOCs) in Livingstone’s stone and Ailsa Craig (2020–2022).

Supplementary Figure S1. Workflow for extraction and GC-MS analysis of free- and glycoside-derived volatile organic compounds (VOCs).

Supplementary Figure S2. Heatmap based on *Z*-scores of annotated compounds for free- and glycoside-derived volatile organic compounds (VOCs).

Supplementary Figure S3. PCA score plots of annotated free- and glycoside-derived volatile organic compounds (VOCs) in the profiles of different tomato cultivars.

Supplementary Data S1. *Z*-scores of free- and glycoside-derived volatile organic compounds (VOCs) in 13 tomato cultivars (2020–2022).
