# Supplementary material for: Effect of climate conditions on the free and glycoside-derived volatile compounds in tomato cultivars
Source: Plant Biotechnol (Tokyo). 2025 Dec 25;42(4):475–83. doi: 10.5511/plantbiotechnology.25.0717a (PMC12781902; doi:10.5511/plantbiotechnology.25.0717a)
Supplement: Supplementary Data [file plantbiotechnology-42-4-25.0717a-s001.pdf]

Supplementary Table S1. List of 13 tomato cultivars used in this study.

Tomato cultivar	Stage	Color	Fruit type	Breed type	Development company
Alisa Craig	Fully ripen	Bright red	Standard	Heirloom	
Brandywine	Fully ripen	Pink	Beefsteak	Heirloom	
Cherokee purple	Fully ripen	Dusky red	Beefsteak	Heirloom	
Chika	Fully ripen	Deep red	Cherry	Hybrid	TAKII & Co., Ltd
Chocolate cherry	Fully ripen	Brick red with deep chocolate	Cherry	Heirloom	
FloraDade	Fully ripen	Bright red	Standard	Fixed	
FL8059	Fully ripen	Red	Standard	Hybrid	
Large red cherry	Fully ripen	Bright red	Cherry	Heirloom	
Lemon Drop	Fully ripen	Yellow	Cherry	Heirloom	
Livingstone's stone	Fully ripen	Red	Standard	Heirloom	
Mexico Midget	Fully ripen	Dark red	Cherry	Heirloom	
Momotaro Fight	Fully ripen	Red	Standard	Hybrid	TAKII & Co., Ltd
Sungold	Fully ripen	Deep orange	Cherry	Heirloom	

Supplementary Table S2. Commonly detected compounds annotated in both free- and glycoside-derived volatile organic compounds (VOCs) across 13 tomato cultivars over three years.

Compound	RI	Chemical class	Molecular Formula	Parental ion (*m/z*)	Top 3 ions (*m/z*)
n-Hexanal	797.8	Aldehyde	$C_6H_{12}O$	100	44, 56, 72
2-Hexenal	845.1	Aldehyde	$C_6H_{10}O$	98	41, 55, 69
(*E*)-2-Hexenol	866.6	Alcohol	$C_6H_{12}O$	100	57, 41, 82
1-Nitro-pentane	899.5	Alkane	$C_5H_{11}NO_2$	117	43, 55, 71
Pentyl-acetate	913.4	Ester	$C_7H_{14}O_2$	130	43, 70, 61
Dimethyl-trisulfide	969	Other	$C_2H_6S_3$	126	126, 79, 45
1-Octen-3-one	976.5	Ketone	$C_8H_{14}O$	126	55, 70, 97
Hexyl acetate	1011.1	Ester	$C_8H_{16}O_2$	144	43, 56, 61
2-Methoxy-phenol	1083.4	Aromatic	$C_7H_8O_2$	124	109, 124, 87
3-(4-Methyl-3-pentenyl)-furan	1097.3	Other	$C_{10}H_{14}O$	150	69, 81, 150
Nonanal	1102.8	Aldehyde	$C_9H_{18}O$	142	41, 57, 70
Methyl octanoate	1121.5	Ester	$C_9H_{18}O_2$	158	74, 87,43
(2*E,6Z*)-Nonadienal	1152.1	Aldehyde	$C_9H_{14}O$	138	41, 70, 27
Octanoic acid	1168.5	Other	$C_8H_{16}O_2$	144	60, 73, 101
Methyl salicylate	1193.5	Aromatic	$C_8H_8O_3$	152	120, 92, 152
Decanal	1203.8	Aldehyde	$C_{10}H_{20}O$	156	41, 57, 29
(*E*)-2-Decenal	1260.1	Aldehyde	$C_{10}H_{18}O$	154	41, 70, 55
(2*E,*4*Z*)-Decadienal	1292.7	Aldehyde	$C_{10}H_{16}O$	152	81, 41, 67
(2*E,*4*E*)-Decadienal	1314.8	Aldehyde	$C_{10}H_{16}O$	152	81, 41, 67
Eugenol	1351.9	Aromatic	$C_{10}H_{12}O_2$	164	164, 77, 103
(*E*)-2-Undecenal	1369.2	Aldehyde	$C_{11}H_{20}O$	168	98, 69, 41
Tetradecane	1397.8	Alkane	$C_{14}H_{30}$	198	57, 43, 71
Dodecanal	1406.8	Aldehyde	$C_{12}H_{24}O$	184	41, 57, 82

RI: Retention indices which were calculated using a series of n-alkane standards (C8–C20).

Top 3 mass: Top three fragment ions (by intensity) are shown for each compound.

Supplementary Table S3. Specific compounds annotated in free VOCs across 13 tomato cultivars over three years.

Compound	Odor threshold (ng/L)	RI	Chemical class	Molecular Formula	Parental ion (*m/z*)	Top 3 ions (*m/z*)	Odor Descriptor	Reference
2-Propoxyethanamine	Unknown	838.1	Other	$C_5H_{13}NO$	103	30, 45, 43	Unknown	
2-Methyl-1-butanol acetate	$3.13*10^5$	877.5	Ester	$C_7H_{14}O$	130	43, 70, 55	Fruity odor like bananas	Cameleyre et al. 2017
Sorbic aldehyde	Unknown	909.4	Aldehyde	C_6H_8O	96	81, 41, 96	Green odor	
3-Ethyl-1,5-octadiene	Unknown	937.7	Alkene	$C_{10}H_{18}$	138	69, 41, 68	Unknown	
6-Methyl-2-heptanone	Unknown	953.8	Ketone	$C_8H_{16}O$	128	43, 58, 71	Green odor	
(*Z*)-2-Heptenal	13	957.7	Aldehyde	$C_7H_{12}O$	112	41, 27, 55	Green, fatty odor	Ozkaya et al. 2018
6-Methyl-5-hepten-2-one	50	984.3	Ketone	$C_8H_{14}O$	126	43, 69, 108	Orange fruity odor	Okan Ozkaya et al. 2018
2-Pentylfuran	6000	989.9	Other	$C_9H_{14}O$	138	81, 82, 53	Green bean, butter odor	Wang et al. 2023
alpha-Terpinene	$1.4*10^6$	998.8	Terpene	$C_{10}H_{16}$	136	93, 121, 136	Lemon odor	
Benzeneaceta-ldehyde	4000	1043.2	Aromatic	C_8H_8O	120	91, 65, 120	Grassy odor	Wang et al. 2023
(*E*)-2-Octenal	3000	1058.1	Aldehyde	$C_8H_{14}O$	126	41, 55, 70	Green-leafy odor	Wang et al. 2023
Levomenthol	2000	1177.3	Terpene	$C_{10}H_{20}O$	156	71, 81, 95	Peppermint odor	OARSWEEL, 2014
beta-Cyclocitral	19000	1221.1	Aldehyde	$C_{10}H_{16}O$	152	109, 137, 67	Sweet-tobacco and grape odor	Xiang et al. 2020
(*Z*)-3,7-Dimethyl-2,6-octadienal	Unknown	1237.3	Aldehyde	$C_{10}H_{16}O$	152	41, 69, 84	Lemon odor	

(*E*)-3,7-Dimethyl-2,6-octadienal	Unknown	1266.2	Aldehyde	$C_{10}H_{16}O$	152	41, 69, 84	Lemon odor
alpha-Copaene	Unknown	1382.6	Alkene	$C_{15}H_{24}$	204	119, 105, 161	Woody odor
(*E*)-6,10-Dimethyl-5,9-undecadien-2-one	Unknown	1438.4	Ketone	$C_{13}H_{22}O$	194	43, 41, 69	Floral odor
Hexadecane	Unknown	1597.7	Alkane	$C_{16}H_{34}$	226	57, 43, 71	Gasoline odor

RI: Retention indices which were calculated using a series of n-alkane standards (C8–C20).

Top 3 mass: Top three fragment ions (by intensity) are shown for each compound.

Supplementary Table S4. Specific compounds annotated in glycoside-derived VOCs across 13 tomato cultivars over three years.

Compound	Odor threshold (ng/L)	RI	Classification	Molecular Formula	Parental ion (*m/z*)	Top 3 ions (*m/z*)	Odor descriptor	Reference
5-Methyl-3-hexanone	Unknown	853.1	Ketone	$C_7H_{14}O$	114	57, 41, 85	Sulfurous odor	
2-Methylbutyl acetate	Unknown	875.4	Ester	$C_7H_{14}O_2$	130	43, 70, 55	Fruity odor	
(2*E*,4*E*)-Hexadienal	$1.8*10^6$	907.2	Aldehyde	C_6H_8O	96	81, 39, 41	Sweet-green citrusy odor	IARC, 2013
3-Nonyne	Unknown	935.7	Other	C_9H_{16}	124	67, 95, 41	Green odor	
(+)-2-Carene	Unknown	982.8	Alkene	$C_{10}H_{16}$	136	93, 121, 136	Sweet and pungent odor	
gamma-Terpinene	$2.6*10^5$	997.4	Terpene	$C_{10}H_{16}$	136	93, 931, 136	Gasoline, Turpentine-like odor	Cometto-Muñiz et al. 1998
1-Octanol	$1.1*10^5$	1056.8	Alcohol	C_8H1_8O	130	56, 41, 55	Waxy odor	Wang et al. 2023
Citronellol	$6.2*10^4$	1176.7	Terpene	$C_{10}H_{20}O$	156	69, 82, 55	Floral odor	Qian et al. 2005
Neral	30	1237	Aldehyde	$C_{10}H_{16}O$	152	69, 94, 41	Lemon odor	Distefano et al. 2022
Geranial	12	1266.1	Aldehyde	$C_{10}H_{16}O$	152	69, 41, 84	Lemon, mint odor	Ozkaya et al. 2018
beta-Cubebene	Unknown	1383	Terpene	$C_{15}H_{24}$	204	161, 105, 91	Citrus, fruity odor	
Neryl acetone	Unknown	1439	Ketone	$C_{13}H_{22}O$	194	43, 69, 41	Fatty odor	

RI: Retention indices which were calculated using a series of n-alkane standards (C8–C20).

Top 3 mass: Top three fragment ions (by intensity) are shown for each compound.

Supplementary Table S5. Main climate conditions from April to July in 2020-2022.

Year	2020				2021				2022			
Month	Apr.	May	Jun.	Jul.	Apr.	May	Jun.	Jul.	Apr.	May	Jun.	Jul.
Max T (℃)	22.9	28.7	29.7	31.1	24.2	27.3	29.7	34.4	25.4	29.6	31.5	33.2
Min T (℃)	4.2	10.5	17.3	18.4	7.3	10.8	16.6	19.9	3.7	10.5	15	21.9
A Max T (℃)	28.1				28.9				29.93			
A Min T (℃)	12.6				13.65				12.78			
DD (h)	224.8	194	146.7	70.8	234.1	175.7	160.7	191.6	179.6	197.1	172.3	195.6
Preci. (mm)	173	68.5	219.5	312.5	119.5	113	115	366	234.5	169.5	96	189.5

Max T: maximum temperature
Min T: minimum temperature
A Max T: Average maximum temperature
A Min T: Average minimum temperature
DD, Daylight duration
Reference: Japan meteorological agency, 2025 (https://www.jma.go.jp/jma/indexe.html)

Supplementary Table S6. Relative peak areas of unique free volatile organic compounds (VOCs) in Livingstone's stone and Ailsa Craig cultivars (2020-2022).

	Livingstone's stone						Alisa Craig					
	2020		2021		2022		2020		2021		2022	
Compounds	Mean	SD	Mean	SD	Mean	SD	Mean	SD	Mean	SD	Mean	SD
Sorbic aldehyde	6.4E-03	2.8E-03	1.2E-02	7.2E-03	1.9E-07	3.6E-07	9.9E-03	2.8E-03	1.2E-02	2.8E-03	2.4E-08	4.3E-08
(*Z*)-2-Heptenal	4.2E-03	5.9E-03	1.4E-04	3.7E-05	6.3E-02	2.4E-02	2.0E-03	2.4E-03	1.1E-04	6.3E-05	5.1E-02	3.3E-02
(*E*)-2-Octenal	1.1E-01	6.7E-02	4.3E-02	2.5E-02	2.5E-01	1.5E-01	1.3E-01	3.5E-02	6.5E-02	7.0E-03	1.6E-01	1.2E-01
Beta-cyclocitral	8.8E-03	1.6E-03	4.1E-03	9.0E-04	1.2E-02	2.7E-03	1.2E-02	2.8E-03	8.5E-03	1.7E-03	5.6E-03	2.2E-03
(*Z*)-3,7-Dimethyl-2,6-octadienal	1.7E-02	1.0E-03	3.3E-03	1.4E-03	6.5E-03	1.7E-03	1.0E-02	1.2E-03	3.8E-03	1.3E-03	2.7E-03	1.4E-03
(*E*)-3,7-Dimethyl-2,6-octadienal	3.8E-02	1.6E-03	1.0E-02	2.6E-03	9.9E-03	2.8E-03	2.6E-02	1.5E-03	9.2E-03	2.7E-03	4.6E-03	2.1E-03
6-Methyl-2-heptanone	3.5E-04	3.9E-04	5.0E-04	3.8E-04	3.1E-08	1.8E-08	7.5E-04	4.3E-04	1.4E-03	6.3E-05	9.3E-08	1.3E-07
6-Methyl-5-hepten-2-one	7.6E-01	1.1E-01	2.6E-01	1.3E-01	4.7E-01	1.4E-01	4.7E-01	5.2E-02	3.1E-01	6.8E-02	2.3E-01	6.7E-02
(*E*)-6,10-Dimethyl 5,9-undecadien-2-one	5.5E-02	6.1E-03	1.4E-02	4.3E-03	0.0E+00	0.0E+00	2.7E-02	3.6E-03	1.1E-02	4.4E-03	2.5E-03	2.2E-03
Alpha-terpinene	1.3E-03	2.0E-03	5.8E-03	3.7E-03	2.4E-02	7.3E-03	1.4E-03	6.1E-04	1.8E-02	9.8E-03	1.5E-04	1.6E-04
Levomenthol	7.8E-04	1.1E-04	3.2E-04	3.0E-04	1.4E-03	1.9E-04	6.6E-04	7.5E-05	2.3E-04	2.0E-04	2.0E-04	1.6E-04
2-Methyl-1-butanol acetate	3.4E-04	3.9E-04	1.3E-03	9.1E-04	6.5E-05	1.8E-05	4.5E-05	5.2E-05	1.3E-03	3.9E-04	9.5E-05	4.3E-05
Hexadecane	1.1E-04	1.0E-04	7.5E-04	6.7E-04	1.6E-08	2.8E-08	1.9E-04	3.4E-05	2.1E-04	3.2E-05	2.2E-08	3.5E-08
3-Ethyl-1,5-octadiene	1.5E-07	2.0E-07	1.2E-06	2.0E-06	2.3E-08	1.8E-08	4.0E-06	6.0E-06	1.9E-04	1.6E-04	2.1E-07	1.9E-07
Alpha-copaene	5.7E-07	9.7E-07	5.7E-03	4.5E-03	5.0E-06	2.5E-06	3.8E-03	4.9E-03	3.8E-03	6.4E-04	1.0E-06	6.1E-07
Benzeneacetaldehyde	2.4E-03	1.0E-03	4.0E-03	1.4E-03	1.2E-03	2.4E-03	3.8E-03	7.7E-05	1.1E-03	3.3E-04	2.2E-04	2.7E-04

2-Propoxyethanamine	1.4E-05	1.3E-05	1.2E-05	1.6E-05	2.5E-06	3.5E-06	4.0E-06	5.0E-06	3.0E-06	2.0E-06	3.4E-06	3.8E-06
2-Pentylfuran	7.7E-02	2.3E-02	5.2E-02	1.8E-02	1.5E-01	6.8E-02	6.9E-02	2.0E-02	5.6E-02	6.5E-03	5.8E-02	1.8E-02

Mean：mean of relative peak area; SD：standard deviation

Supplementary Table S7. Relative peak areas of unique glycoside-derived volatile organic compounds (VOCs) in Livingstone's stone and Ailsa Craig (2020-2022).

	Livingstone's stone						Alisa Craig					
	2020		2021		2022		2020		2021		2022	
	Mean	SD	Mean	SD	Mean	SD	Mean	SD	Mean	SD	Mean	SD
(2*E*,4*E*)-Hexadienal	5.6E-05	2.8E-03	2.3E-04	2.3E-04	1.2E-04	1.2E-04	3.6E-04	2.8E-03	2.2E-04	7.7E-05	4.7E-05	4.4E-05
Neral	1.4E-01	3.5E-01	4.5E-09	7.6E-09	4.6E-03	1.3E-02	4.5E-03	8.3E-03	1.4E-08	3.2E-07	1.5E-08	5.4E-07
Geranial	9.1E-05	2.2E-04	4.9E-04	2.9E-04	6.4E-04	1.1E-03	5.4E-08	1.3E-07	9.4E-04	3.3E-04	2.8E-04	2.3E-04
5-Methyl-3-hexanone	1.2E-04	7.9E-05	2.0E-04	4.1E-05	2.5E-04	2.2E-04	6.2E-06	2.0E-06	5.4E-05	2.5E-05	1.5E-04	1.0E-04
Neryl acetone	1.1E-05	1.9E-05	1.7E-07	1.4E-07	1.9E-07	2.3E-07	8.4E-05	5.9E-05	1.9E-07	1.5E-07	3.8E-08	4.3E-08
Gamma-terpinene	5.3E-05	6.9E-05	2.2E-04	2.9E-04	7.9E-04	8.0E-04	7.0E-05	1.0E-04	1.7E-04	1.8E-04	5.1E-04	6.2E-04
Citronellol	2.1E-04	5.1E-04	9.6E-06	1.5E-05	4.5E-06	9.3E-06	2.6E-05	1.9E-05	2.0E-08	4.2E-08	1.3E-07	1.9E-07
Beta-cubebene	5.6E-05	1.4E-04	2.7E-06	8.9E-07	1.4E-06	2.5E-06	2.0E-07	1.9E-07	1.2E-06	7.2E-07	3.8E-07	2.9E-07
2-Methylbutyl acetate	4.9E-05	4.3E-05	2.9E-04	1.9E-04	8.4E-05	4.0E-05	2.7E-06	2.0E-06	7.6E-05	5.1E-05	2.6E-05	2.2E-05
(+)-2-Carene	1.4E-05	3.5E-05	8.1E-03	8.7E-03	2.7E-07	4.1E-07	1.4E-03	1.5E-03	1.1E-03	1.1E-03	2.1E-06	1.9E-06
3-Nonyne	2.8E-03	4.4E-03	2.6E-03	3.3E-03	3.9E-03	4.4E-03	1.9E-03	2.3E-03	2.2E-03	2.4E-03	1.1E-03	1.4E-03
1-Octanol	1.6E-05	2.1E-05	1.4E-04	8.5E-05	4.6E-07	3.6E-07	1.9E-05	2.1E-05	3.9E-08	3.1E-08	2.9E-07	2.3E-07

Mean：mean of relative peak area; SD：standard deviation

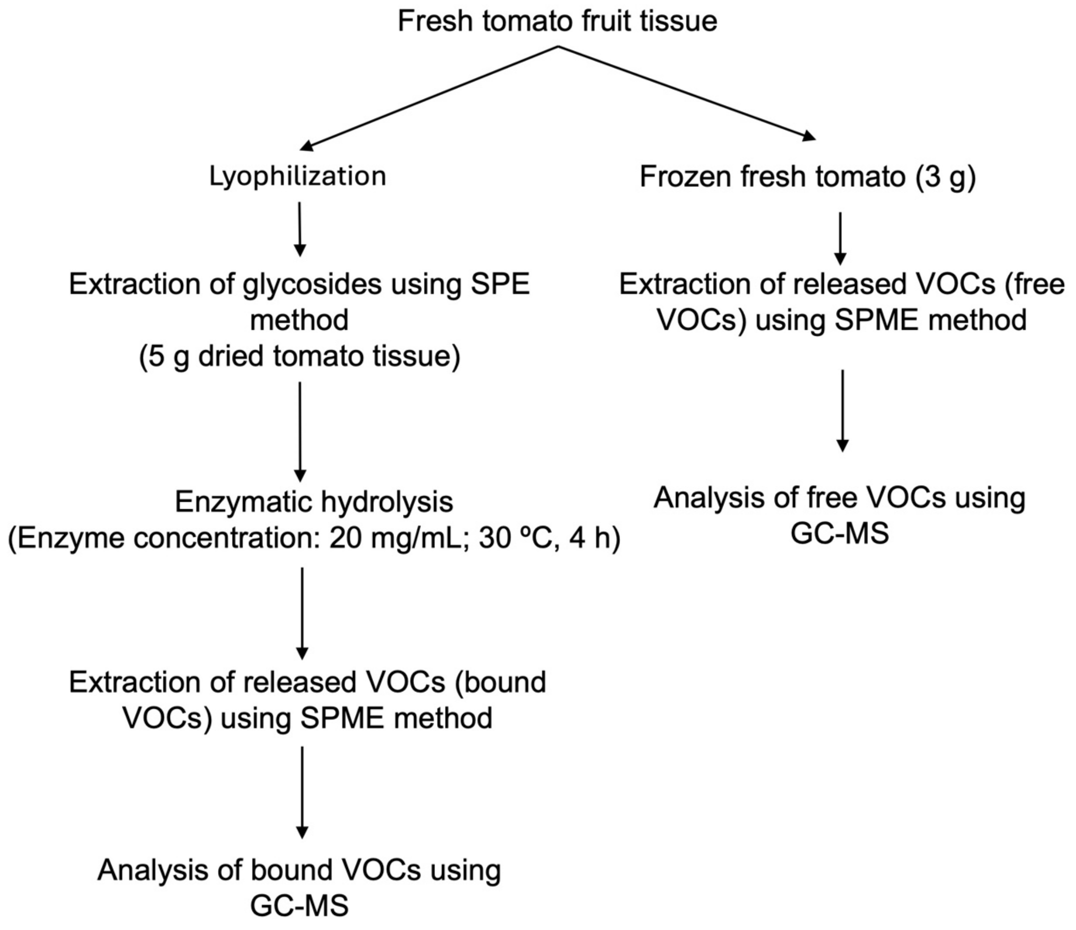

Supplementary Figure S1. Workflow for extraction and GC-MS analysis of free- and glycoside-derived volatile organic compounds (VOCs).

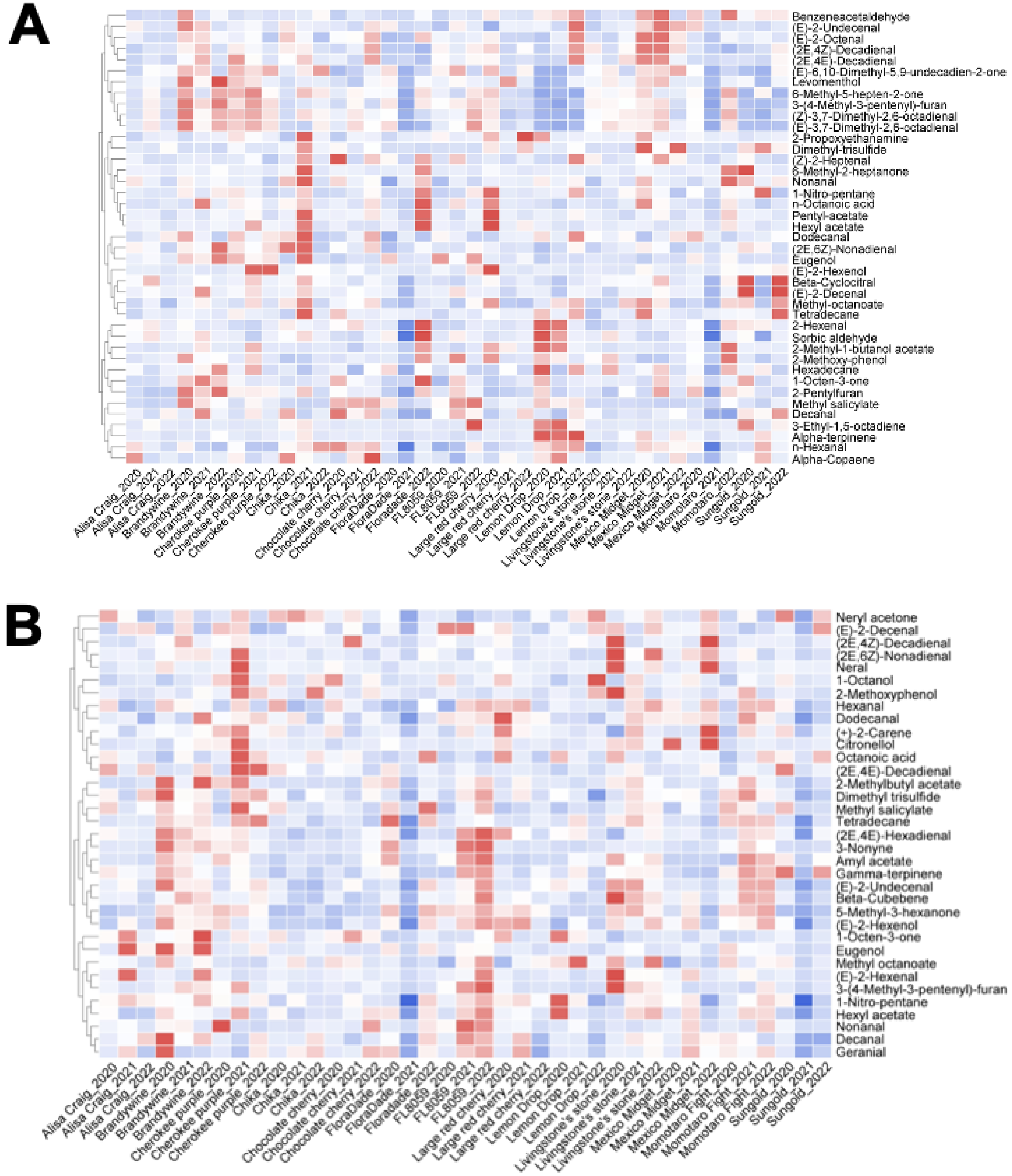

Supplementary Figure S2. Heatmap based on *Z*-scores of annotated compounds for free- (A) and glycoside-derived (B) volatile organic compounds (VOCs). Data were obtained from GC-MS analysis of tomato samples grown in different years (n = 3-4). Color scale represents the normalized abundance of each compound across samples, displayed as *Z*-scores. Red indicates higher values, while blue indicates lower values.

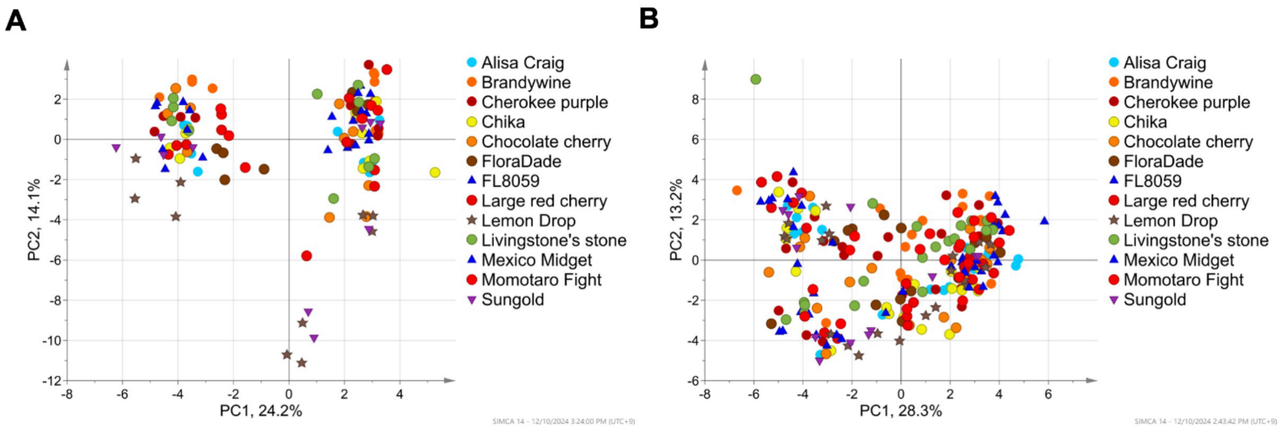

Supplementary Figure S3. PCA score plots of annotated free- (A) and glycoside-derived (B) volatile organic compounds (VOCs) in the profiles of different tomato cultivars. Data were obtained from GC-MS analysis of tomato samples grown in different years (*n* = 3–4).

References

Cameleyre M, Lytra G, Tempere S, Barbe JC (2017) 2-Methylbutyl acetate in wines: Enantiomeric distribution and sensory impact on red wine fruity aroma. *Food Chem* 237: 364–371

Cometto-Muñiz JE, Cain WS, Abraham MH, Kumarsingh R (1998) Trigeminal and olfactory chemosensory impact of selected terpenes. *Pharmacol Biochem Behav* 60: 765–770

Distefano M, Mauro RP, Page D, Giuffrida F, Bertin N, Leonardi C (2022) Aroma volatiles in tomato fruits: The role of genetic, preharvest and postharvest factors. *Agronomy* 12: 1134

IARC Working Group on the Evaluation of Carcinogenic Risks to Humans (2013) Some chemicals present in industrial and consumer products, food and drinking-water. *IARC Monogr Eval Carcinog Risks Hum* 101: 9–549

Japan Meteorological Agency (2020) A site containing information on the climate of Japan. URL: https://www.jma.go.jp/jma/indexe.html (accessed Jun 1, 2025)

OARSWEEL (2014) An online platform for the exchange of information on chemical stressor exposure guidelines, methods for improving occupational risk assessment, and training opportunities. URL: https://www.tera.org/OARS/index.html (accessed Jun 11, 2025)

Ozkaya O, Sen K, Aubert C, Dundar O, Gunata Z (2018) Characterization of the free and glycosidically bound aroma potential of two important tomato cultivars grown in Turkey. *J Food Sci Technol* 55: 4440–4449

Qian MC, Wang Y (2005) Seasonal variation of volatile composition and odor activity value of 'Marion' (*Rubus* spp. hyb) and 'Thornless Evergreen' (*R. laciniatus* L.) blackberries. *J Food Sci* 70: C13–C20

Wang S, Chen H, Sun J, Zhang N, Wang S, Sun B (2023) Effects of cooking methods on aroma formation in pork: A comprehensive review. *Food Chem X* 20: 100884

Xiang H, Shao Y, Gao N, Lu X, An N, Chu W (2020) Removal of β-cyclocitral by UV/persulfate and UV/chlorine process: Degradation kinetics and DBPs formation. *Chem Eng J* 382: 122933
